# Annotations

**Published:** 1909-01-16

**Authors:** 


### ANNOTATIONS.

### The Bombay Medical Congress.

Last year we announced that arrangements were being made for an Indian Medical Congress to be held at Bombay early in 1909. The Congress will begin on February 22, when the Governor, Sir George Clarke, will deliver an inaugural presidential address, and the proceedings will continue on the four following days. In connection with the Congress there will be an exhibition of medical, surgical, and sanitary appliances, etc. It is expected that the Congress will afford opportunities for the exchange of medical views upon recent advances in our knowledge of tropical diseases, and that new and improved methods of treatment may thereby become disseminated. The attendance is expected to number about 2,000, and there is every prospect of a very successful gathering. Many leading authorities from England and elsewhere have promised to contribute papers, and if possible to read them in person. The subjects for discussion are so numerous that it will be necessary for them to be considered in five separate sections. An appropriately designed commemorative medal, so we learn, is to be struck by the Bombay Mint; and this will form an interesting souvenir of what promises to be a valuable Congress upon Indian medicine.

### Medico-legal Aspects of Skiagraphy.

The introduction of *x*-rays has, as members of the profession in all countries have been made aware, entirely altered the possibility of diagnosing certain injuries and of estimating the results of their treatment. A large crop of medico-legal cases has sprung from this revolutionary invention, many of which were decided before surgical opinion had fully settled exactly what are the limitations of skiagraphy. We know now that a fractured bone may unite in such a way as to appear surgically very imperfect when seen by *x*-rays, and yet the functional result be as satisfactory as possible. On the other hand, a bad functional result may not be due to indifferent coaptation of the bone-ends or to malunion, but to other causes altogether unconnected with the bony parts; and such results may be entirely beyond amelioration by any known method in certain cases. These points have been gradually thrashed out, sometimes in law courts where individual members of the profession have on occasions been harshly dealt with. In Germany a novel action for *malapraxis* has recently been decided: a needle was demonstrated by *x*-rays in a child's stomach, but three days later it could not be found by laparotomy. The surgeon was sued for performing an unnecessary operation, but was acquitted: another examination after the operation showed that the needle had travelled further along the alimentary canal. The moral of this case is one worth bearing in mind: it is that only quite recent skiagrams are of value in locating a foreign body. One does not feel at all confident that the defendant would have got a verdict in this country in similar circumstances. Another point never to be forgotten in treating any injury to a bone or joint, the nature of which is in the very least obscure, is that an action for negligence may follow the non-recognition of any gross lesion which could have been detected by the employment of *x*-rays. Hæmatoma, swelling, pain, and so on have been accepted in courts of law in the past as excuses for missing such lesions, but the case is very different now: failure to advise *x*-rays to these patients will almost certainly be construed as *malapraxis* in the British courts.

### Alcohol as a Drug.

There are few of the more potent and commonly ordered drugs about which there are such wide differences of opinion among practitioners of medicine as about alcohol. In this country, speaking broadly, the large majority employ it in various forms as one of their stock measures in the treatment of certain acute and very exhausting diseases, as, for example, pneumonia. But there is a minority, and one which numbers some very eminent men, which refuses to order alcohol in these circumstances; holding that it has serious disadvantages from which other drugs are free. On both sides are ranged men of the highest reputation, both as clinicians and as laboratory workers: the manifestoes which have been issued in this country have been evidence of this. So far the division of opinion is simply over a matter of therapeutics, the merits of a particular drug: but the question has much wider aspects of a sociological nature, which cannot be ignored. How far the moderate use of alcoholic drinks as beverages is harmful or beneficial is a point perhaps midway between the strictly medical and the strictly economic sides of the problem: upon this unanimity has no more been obtained than upon its use for acute diseases, though here again the majority are opposed to total abstinence. Physicians cannot, unfortunately, exclude the consideration of drunkenness and its attendant evils, for their recommendations of alcohol have been known to be the origin of the alcohol habit. In Germany the Society of Physicians for Abstinence is very active, and, though only a small percentage of German practitioners have as yet been converted, its membership is steadily increasing. Our own views on the physiological results of strictly moderate alcoholic indulgence are well known: they are based upon careful chemical and physiological experiment, as detailed by the Commissions on Whiskies and on Wines, appointed by The Hospital, whose reports have been published in these columns (April 7, 1906, and June 15, 1907, respectively). It is, of course, quite possible without inconsistency to accept the conclusions which we then formulated and yet to hold that the example of total abstinence is imperative for the sake of the weaker vessels. We notice, however, that most of the adherents of the anti-alcohol cause will admit nothing but evil of the most minute quantity of alcohol, in whatever form; and it is quite probable that it is this extreme and prejudiced attitude which accounts for the lukewarm support which the medical profession has yet accorded them.

